# Comparative Evaluation of Modern Disinfection Methods on Smear Layer Removal and Bond Strength to Caries-Affected Dentin: A KTP Laser, Nanocare Gold, Photodynamic Therapy Study

**DOI:** 10.12669/pjms.42.2.14232

**Published:** 2026-02

**Authors:** Abdulaziz A. Al-Kheraif, Hanan Alsunbul, Hind Alhaidry, Aftab Ahmed Khan

**Affiliations:** 1 Abdulaziz A. Al-Kheraif Development and Innovation in Oral and Dental Health Research Chair, Department of Dental Health, College of Applied Medical Sciences, King Saud University, Riyadh, Saudi Arabia; 2 Hanan Alsunbul Department of Restorative Dentistry, College of Dentistry, King Saud University, Riyadh, Saudi Arabia; 3 Hind Alhaidry Advanced General Dentistry, Prince Sultan Military Medical City, Riyadh, Saudi Arabia; 4 Aftab Ahmed Khan Development and Innovation in Oral and Dental Health Research Chair, Department of Dental Health, College of Applied Medical Sciences, King Saud University, Riyadh, Saudi Arabia

**Keywords:** Chlorhexidine, Photodynamic therapy, Potassium titanyl phosphate, Nanocare Gold, Caries-affected dentin, Smear layer

## Abstract

**Objective:**

To assess the efficacy of four contemporary cavity disinfection protocols-potassium titanyl phosphate (KTP) laser, nanosilver-gold nanoparticle solution (Nanocare Gold), methylene blue-mediated photodynamic therapy (MB-PDT), and chlorhexidine gluconate (CHX)-on smear layer (SL) removal and shear bond strength (SBS) of fifth-generation adhesive bonded to caries-affected dentin (CAD).

**Methodology:**

This in vitro experimental study was approved by the Institutional Review Board of King Saud University from July 1st, 2025, to 30th December 2025. Sixty extracted human mandibular molars with carious lesions extending into the middle third of dentin were collected. Following caries removal and surface standardization, specimens were randomly allocated into five groups: Group-I (control, no disinfection), Group-II (2% CHX gel), Group-III (KTP laser, 532 nm, 1 W), Group-IV (MB-PDT with diode laser activation at 638 nm), and Group-V (Nanocare Gold). SL removal was assessed using scanning electron microscopy according to Hülsmann’s criteria. Following fifth-generation adhesive application and composite restoration, specimens underwent thermocycling. SBS was measured using a universal testing machine, and failure modes were analyzed stereo-microscopically. Statistical analysis employed one-way ANOVA with post hoc Tukey test (α=0.05).

**Results:**

KTP laser demonstrated superior SL removal (1.26±0.032) and the highest bond strength (9.10±1.02 MPa), statistically comparable to Nanocare Gold (1.34±0.044; 8.94±0.97 MPa, p>0.05). MB-PDT showed minimal SL elimination (3.54±0.065) and the lowest bond strength (6.89±0.78 MPa), similar to CHX (p>0.05).

**Conclusion:**

KTP laser and Nanocare Gold demonstrated optimal performance for cavity disinfection, effectively removing the smear layer while enhancing adhesive bond strength to CAD.

## INTRODUCTION

Dental caries remains one of the most prevalent chronic diseases worldwide. Management requires selective removal of infected dentin while preserving caries-affected dentin (CAD) to maintain pulpal vitality and structural integrity-a fundamental principle of minimal intervention dentistry.^1^,^2^ Effective cavity disinfection is essential for eliminating residual bacteria and optimizing restoration longevity. Cavity preparation generates a smear layer (SL).^3^ This 0.5-2 μm layer compromises micromechanical interlocking between adhesive systems and dentin, reducing bond strength and restoration durability.^4^ An ideal cavity disinfectant should achieve microbial elimination while facilitating SL removal to optimize adhesive bonding.^4^

Chlorhexidine gluconate (CHX), a bis-biguanide antiseptic, has long been advocated as a cavity disinfectant due to its broad-spectrum antimicrobial activity and ability to inhibit matrix metalloproteinases responsible for hybrid layer (HL) degradation.^5^ However, conflicting evidence exists regarding its impact on bond strength, with several studies reporting decreased adhesive performance.^6^,^7^ Furthermore, its SL removal efficacy remains inferior to that of chelating agents. Potassium titanyl phosphate (KTP) laser, operating at 532 nm, has garnered attention for its photothermal effects on dental hard tissues. Laboratory studies demonstrate the KTP laser’s capability to reduce demineralization by 30-50% through morphological alterations.^2^,^8^

However, the comprehensive evaluation of its effects on SL elimination and adhesive bond strength to CAD remains limited. Photodynamic therapy (PDT) exploits photochemical reactions between photosensitizers, light, and oxygen to generate cytotoxic reactive oxygen species.^9^ Methylene blue (MB), with maximum absorption at 665 nm, offers the advantages of low cost and effectiveness against diverse microbial species.^10^ However, contradictory findings regarding its effects on adhesive bonding warrant further investigation.

Nanocare Gold, a colloidal suspension containing silver and gold nanoparticles, has been proposed as a cavity disinfectant. Silver nanoparticles exert antimicrobial effects through multiple mechanisms, while gold nanoparticles enhance collagen stability through cross-linking induction.^11^ Preliminary investigations suggest comparable bond strength to CHX, though systematic evaluation of SL removal efficacy remains absent. The present investigation compared contemporary cavity disinfection protocols’ effects on SL elimination and adhesive bonding to CAD. The null hypotheses tested were: (1) no significant differences would exist in SL removal efficacy among treatments; and (2) SBS would not differ significantly among disinfection protocols.

## METHODOLOGY

### Specimen Selection and Preparation:

Sixty freshly extracted human mandibular first permanent molars were collected from patients aged 18-45 years. This is an in vitro experimental study.

### Ethical Approval:

It was obtained from the Institutional Review Board of King Saud University FC-41-2025, July 1st, 2025, to 30th December 2025.

### Inclusion criteria:

Presence of occlusal or proximal carious lesions confirmed radiographically to extend into the middle third of dentin, absence of previous restorations, no cracks or structural defects, and absence of enamel hypoplasia or fluorosis.^10^ Soft tissue remnants, calculus, and organic debris were mechanically removed using an ultrasonic scaler. Specimens underwent thorough disinfection in 0.5% Chloramine-T-trihydrate solution.^2^ Each tooth was embedded vertically in self-curing acrylic resin upto cementoenamel junction using a custom-fabricated silicone mold. CAD was identified using the caries detector dye method as described by Nakajima et al.^12^ The occlusal surface was reduced using a water-cooled low-speed diamond saw operating at 350 rpm to expose a flat CAD surface perpendicular to the tooth axis. To standardize the SL thickness and composition across all specimens, exposed CAD surfaces were finished using 600-grit silicon carbide abrasive paper (Carbimet, Buehler) under water irrigation for 60 seconds with standardized hand pressure.

### Experimental Groups and Disinfection Protocols:

Following standardized SL creation, specimens were randomly allocated into five experimental groups (n=12 per group)

### Group-I (Control - No Disinfection):

CAD surfaces received no disinfection treatment and were maintained in humid conditions until adhesive application.

### Group-II (Chlorhexidine Gluconate):

A 2% chlorhexidine gluconate gel was applied to the CAD surface using a sterile microbrush applicator, ensuring complete coverage. Following a 20-second contact time as per manufacturer recommendations, cavities were thoroughly rinsed with distilled water for 10 seconds and gently air-dried for five seconds to maintain optimal dentin moisture without desiccation.^13^

### Group-III (KTP Laser):

Specimens were irradiated using a KTP laser system (Fotona Fidelis Plus III, Fotona Ljubljana, Slovenia) with the following parameters: wavelength 532 nm (green light), continuous wave mode, output power 1.0 W, spot size 1 mm diameter, energy density 127.3 J/cm^2^. The fiber optic delivery system was positioned perpendicular to the CAD surface at a fixed working distance of 1 mm using a custom-designed positioning jig. Four consecutive irradiation cycles were performed, each consisting of 10 seconds of laser exposure followed by a 5-second rest period.^2^

### Group-IV (Methylene Blue-Mediated Photodynamic Therapy):

A 0.01% (100 mg/L) methylene blue solution (Methylthioninium chloride, Merck KGaA, Darmstadt, Germany) in sterile 0.9% saline was applied to the CAD surface. The photosensitizer had a five-minutes pre-irradiation incubation in darkness. Photoactivation used a diode laser (SiroLaser Blue, Dentsply Sirona, Bensheim, Germany) with parameters: wavelength 638 nm, continuous wave mode, output power 1.5 W, fiber optic diameter 400 μm, irradiation time 30 seconds. The fiber optic tip was kept 2 mm from the dentin surface and moved in circular motions for uniform energy distribution across the treatment area.^14^

### Group-V (Nanocare Gold):

Nanocare Gold solution (Dental Nanotechnology SA, Morges, Switzerland), containing silver nanoparticles (10-80 nm) and gold nanoparticles (5-20 nm) in colloidal suspension, was applied to the CAD surface using the manufacturer-provided disposable applicator tip. Five drops (approximately 0.25 mL) were dispensed to ensure complete surface coverage. The solution was allowed to air-dry for three minutes at room temperature to facilitate nanoparticle deposition and interaction with the dentin substrate. No rinsing was performed following the drying period as per the manufacturer’s instructions.^15^ Following disinfection protocol application, all specimens were stored in artificial saliva (Biotène Oral Balance, GlaxoSmithKline, Brentford, UK) at 37°C.

### SEM evaluation of SL:

Two specimens from each experimental group were selected for scanning electron microscopy (SEM) analysis of SL removal. Dentin discs (1.0 mm thickness) were sectioned from the CAD surface. Specimens were mounted on aluminum stubs using carbon adhesive and sputter-coated with a 10-nm gold layer under vacuum. SL presence and dentinal tubule patency were evaluated using the five-point scoring system described by Hülsmann et al.^16^ Two calibrated, blinded evaluators independently scored each image, resolving disagreements through consensus.

### Adhesive Bonding and Composite Restoration (n=10):

The prepared CAD surfaces underwent acid etching using 37% orthophosphoric acid gel applied for 15 seconds, followed by thorough water rinsing for an equal duration and brief air-drying (five seconds) to achieve appropriate surface wetness. The bonding procedure utilized a fifth-generation total-etch adhesive (Adper Single Bond 2, 3M ESPE, St. Paul, MN, USA) applied in two successive layers. Excess adhesive material was dispersed using gentle air-blowing for five seconds. To volatilize the solvent carrier, the bonding agent was subsequently polymerized for 10 seconds using a light-emitting diode curing device operating at 1,000 mW/cm^2^ output intensity.^2^ Standardized composite resin cylinders were fabricated using a nano-hybrid resin composite (Filtek Z350 XT, shade A2, 3M ESPE) placed incrementally in 2-mm layers into a transparent polyethylene Tygon tube (Saint-Gobain Performance Plastics, Akron, OH, USA) with 3-mm internal diameter and 3-mm height. Each increment was light-cured for 20 seconds.

### Artificial Aging Protocol:

To simulate intraoral aging conditions and thermal stress, bonded specimens underwent thermocycling (THE-1100, SD Mechatronik GmbH, Feldkirchen-Westerham, Germany). The protocol consisted of 10,000 thermal cycles between water baths maintained at 5°C and 55°C. Each cycle comprised 30 seconds of immersion in cold water, followed by five seconds of transfer time, then 30 seconds in hot water.

### SBS Testing:

Following thermocycling, specimens were mounted in a custom-designed jig attached to the lower crosshead of a universal testing machine (Model 3345, Instron Corporation, Norwood, MA, USA). A knife-edge shear blade (0.5 mm thickness) was positioned at the composite-dentin interface. Shear force was applied at a crosshead speed of 1 mm/min until bond failure occurred.

### Failure Mode Analysis:

Following bond strength testing, all debonded surfaces were examined using a stereomicroscope at 40× magnification. Failure modes were classified into three categories based on the pattern of bond failure: (1) adhesive failure, (2) cohesive failure, and (3) mixed failure.

### Statistical Analysis:

All statistical analyses were performed using IBM SPSS Statistics for Windows, version 28.0 (IBM Corporation, Armonk, NY, USA). Data normality was assessed using the Shapiro-Wilk test. ANOVA was employed to compare mean SBS and SL values among the five experimental groups. When significant differences were detected (p<0.05), post hoc pairwise comparisons were conducted using Tukey’s Honestly Significant Difference (HSD) test to identify specific group differences.

## RESULTS

### SL removal assessment:

**Fig.1:**
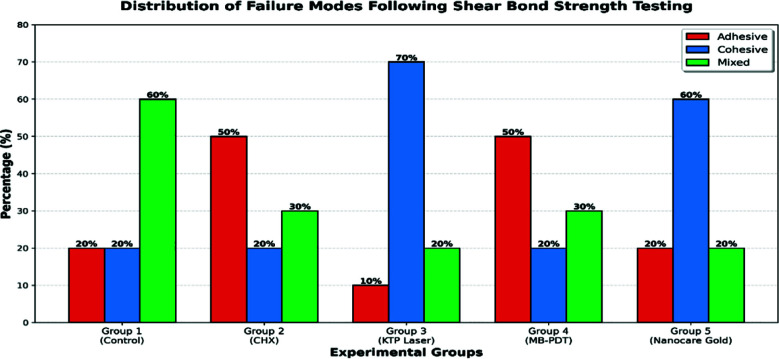
Distribution of fracture pattern analysis.

The extent of smear layer removal from the CAD surface following the application of various surface disinfectants is presented in Table-I. The maximum SL elimination was identified in Group-III (KTP Laser) (1.26±0.032) samples. Whereas the minimum SL removing efficiency was displayed by Group-IV (MB mediated PDT) (3.54±0.065). Intergroup comparative analysis presented that Group-II (CHX) (3.22±0.054) and Group-IV exhibited comparable scores of SL removal. (p>0.05) Likewise, in Group-III and Group-V (Nanocare Gold) (1.34 ± 0.044), no significant variation was observed among the samples regarding their smear layer elimination effectiveness (p>0.05). On the other hand, Group-I (No disinfection) (2.36 ± 0.021) displayed significantly different scores from other tested groups. (p <0.05) (Fig.1).

**Table-I:** SL removal Efficacy from CAD after different surface disinfectants.

Surface Disinfectant Regimes	Mean ± SD (µm)	p-value*
Group-I: No disinfection	2.36 ± 0.021^c^	<0.05
Group-II: CHX	3.22 ± 0.054^b^	
Group-III: KTP Laser	1.26 ± 0.032^a^	
Group-IV: MB mediated PDT	3.54 ± 0.065^b^	
Group-V: Nanocare Gold	1.34 ± 0.045^a^	

* ANOVA p<0.05. Potassium titanyl phosphate (KTP) laser, Chlorhexidine gluconate (CHX), Photodynamic therapy (PDT), Methylene blue. Different superscript lowercase letters indicate statistically significant differences among values within the same column, p<0.05 (Post Hoc Tukey).

### SBS analysis:

Table-II illustrates the SBS values for 5th-generation adhesive systems applied to CAD surfaces after treatment with different disinfecting agents. The maximum SBS was identified in Group-III (KTP Laser) (9.10 ± 1.02 MPa) samples. Whereas minimum bond integrity was displayed by Group-IV (MB mediated PDT) (6.89 ± 0.78 MPa). Intergroup comparison analysis presented that Group-III and Group-V (Nanocare gold) (8.94 ± 0.97 MPa) exhibited comparable scores of adhesion strength. (p>0.05) Likewise, Group-II (CHX) (7.08 ± 0.93 MPa) and Group-IV specimens also demonstrated no significant difference in their SBS outcomes. (p>0.05) On the other hand, Group-I (No disinfection) (7.76 ± 0.59 MPa) displayed significantly different scores compared to the other experimental groups. (p <0.05).

**Table-II:** Evaluation of SBS of adhesive bonded to CAD after using different surface disinfectants.

Tested groups	Mean ± SD (MPa)	p-value*
Group-I: No disinfection	7.76 ± 0.59^c^	<0.05
Group-II: CHX	7.08 ± 0.93^b^	
Group-III: KTP Laser	9.10 ± 1.02^a^	
Group-IV: MB mediated PDT	6.89 ± 0.78^b^	
Group-V: Nanocare gold	8.94 ± 0.97^a^	

* ANOVA p<0.05.Potassium titanyl phosphate (KTP) laser, Chlorhexidine gluconate (CHX), Photodynamic therapy (PDT), Methylene blue. Different superscript lowercase letters indicate statistically significant differences among values within the same column, p<0.05 (Post Hoc Tukey).

### Fracture mode analysis:

**Fig.2A:**
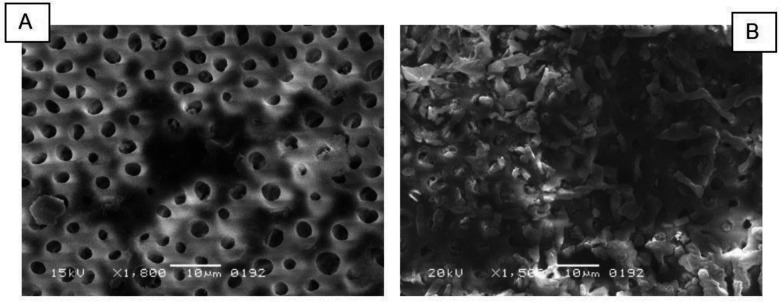
SEM image depicts CAD surface treated with KTP Laser and Nanocare Gold showing different open wide tubular network with no smear layer on the surface. Similarly, Fig.2B: Shows heavy SL on the surface with few or no dentinal openings when CAD was treated with MB mediated PDT and CHX.

Failure mode analysis showed differences in fracture patterns among experimental groups. Groups 3 (KTP laser) and 5 (Nanocare Gold) exhibited mainly cohesive failures. Groups 2 (CHX) and 4 (MB-PDT) showed predominantly adhesive failures. Group-I (control) displayed a balanced distribution with mixed failure (50%) being the most common, indicating intermediate bonding performance (Fig.2).

## DISCUSSION

The present investigation evaluated four contemporary cavity disinfection protocols regarding their dual effects on SL removal and adhesive bonding to CAD. The null hypotheses were rejected, as significant differences were observed among disinfection methods in both SL elimination efficacy and SBS outcomes.

The superior performance of KTP laser irradiation corroborates previous investigations examining laser-dentin interactions.^17^ The 532 nm wavelength exhibits selective absorption by organic matrix components and water molecules within dentinal tubules, inducing localized photothermal effects.^2^ These thermal interactions result in protein denaturation, organic debris vaporization, and localized melting of intertubular dentin surface, effectively removing SL while creating micromechanical irregularities conducive to adhesive penetration.^18^ Similar findings were reported by Machida et al., who demonstrated effective debris and SL removal from root canal surfaces.^19^ The pulsed protocol with rest intervals employed in this study maintained temperature elevation below 10°C, contributing to favorable outcomes without thermal damage.

The comparable efficacy demonstrated by Nanocare Gold to the KTP laser represents a significant finding. The nanosilver-gold formulation likely exerts beneficial effects through multiple mechanisms. Silver nanoparticles (10-80nm) possess high surface area-to-volume ratios, enabling enhanced antimicrobial activity and facilitating penetration into dentinal tubules, disrupting SL structure and creating channels for adhesive infiltration.^20^ Gold nanoparticles interact with collagen fibrils, inducing cross-linking that enhances structural stability and resistance to enzymatic degradation.^15^ SEM observations revealed nanoparticle deposits within tubule orifices, supporting deep penetration and substrate modification hypotheses.^15^ These findings align with AlFawaz et al., who reported comparable bond strength between Nanocare Gold and CHX.^21^

The reduced bonding performance observed with CHX and MB-PDT requires careful interpretation. For CHX, residual organic compounds may interfere with acid etching effectiveness, CHX molecules may occupy bonding sites on collagen fibrils, competing with adhesive monomers, and incomplete removal may create hydrophilic barriers impeding resin infiltration. The timing of CHX application (pre-etching in this study) may have compromised etching efficacy.^22^ Alternative protocols applying CHX after etching might yield different outcomes. MB-PDT’s disappointing performance warrants detailed consideration.

Multiple mechanisms likely contribute MB’s hydrophilic molecular structure facilitates water absorption, interfering with hydrophobic resin infiltration; reactive oxygen species may induce oxidative collagen damage; photosensitizer precipitation creates physical barriers; and singlet oxygen may react with calcium and phosphate, forming precipitates occluding tubules.^10^,^23^ SEM observations of crystalline precipitates support this hypothesis. These findings contrast with AlSunbul et al., who reported enhanced SBS following MB-PDT on sound dentin; this discrepancy may reflect CAD’s altered collagen structure and increased water content, exacerbating MB’s hydrophilic interference effects.^24^,^25^

### Limitations:

The in vitro design cannot replicate oral cavity complexity, including salivary proteins, pellicle formation, and pulpal fluid dynamics. Brief aging (10,000 thermocycles) approximates one year; extended periods would provide more relevant data. Evaluation was limited to one adhesive system; outcomes might differ with other chemistries. Only specific laser parameters and photosensitizer concentrations were tested; optimization studies might identify improved protocols.

### Clinical Innovation and Application:

Cavity disinfection protocol selection influences antimicrobial efficacy and adhesive bonding success. The superior performance of the KTP laser and Nanocare Gold suggests these modalities are viable alternatives to conventional CHX when optimizing adhesive bonding to CAD. For practitioners with KTP laser access, the protocol (532 nm, 1W, pulsed mode) effectively removed SL while enhancing bond strength. For practitioners seeking laser alternatives, Nanocare Gold offers enhanced performance through topical application. The protocol integrates into workflows, though higher material cost versus CHX may influence adoption in resource-limited settings.

## CONCLUSION

KTP laser demonstrated superior smear layer removal and bond strength enhancement, representing an optimal cavity disinfection protocol. Nanocare Gold exhibited comparable performance to the KTP laser, offering a promising alternative without specialized equipment requirements.
